# Diseased and healthy murine local lung strains evaluated using digital image correlation

**DOI:** 10.1038/s41598-023-31345-w

**Published:** 2023-03-20

**Authors:** T. M. Nelson, K. A. M. Quiros, E. C. Dominguez, A. Ulu, T. M. Nordgren, M. Eskandari

**Affiliations:** 1 Department of Mechanical Engineering, University of California, Riverside, CA USA; 2 Division of Biomedical Sciences, Riverside School of Medicine, University of California, Riverside, CA USA; 3 Environmental Toxicology Graduate Program, University of California Riverside, Riverside, CA USA; 4 BREATHE Center, School of Medicine, University of California, Riverside, CA USA; 5 Department of Environmental and Radiological Health Sciences, Colorado State University, Fort Collins, CO USA; 6 Department of Bioengineering, University of California, Riverside, CA USA

**Keywords:** Tissues, Chronic obstructive pulmonary disease, Biomedical engineering, Mechanical engineering, Respiration

## Abstract

Tissue remodeling in pulmonary disease irreversibly alters lung functionality and impacts quality of life. Mechanical ventilation is amongst the few pulmonary interventions to aid respiration, but can be harmful or fatal, inducing excessive regional (i.e., local) lung strains. Previous studies have advanced understanding of diseased global-level lung response under ventilation, but do not adequately capture the critical local-level response. Here, we pair a custom-designed pressure–volume ventilator with new applications of digital image correlation, to directly assess regional strains in the fibrosis-induced ex-vivo mouse lung, analyzed via regions of interest. We discuss differences between diseased and healthy lung mechanics, such as distensibility, heterogeneity, anisotropy, alveolar recruitment, and rate dependencies. Notably, we compare local and global compliance between diseased and healthy states by assessing the evolution of pressure-strain and pressure–volume curves resulting from various ventilation volumes and rates. We find fibrotic lungs are less-distensible, with altered recruitment behaviors and regional strains, and exhibit disparate behaviors between local and global compliance. Moreover, these diseased characteristics show volume-dependence and rate trends. Ultimately, we demonstrate how fibrotic lungs may be particularly susceptible to damage when contrasted to the strain patterns of healthy counterparts, helping to advance understanding of how ventilator induced lung injury develops.

## Introduction

Lung diseases are widely prevalent and detrimental to health, ranking as one of the top causes of death globally^1–3^. Each manifestation of pulmonary illness presents different challenges for proper clinical care. This study investigates the lung mechanics of fibrosis, a harmful condition with a 2–4 year median duration of survival post-diagnosis^4^. Fibrosis may arise from a multitude of factors, such as occupational hazards or genetic predispositions^5,6^. Pathologically, fibrosis is characterized by the deposition of collagen, a relatively inextensible fiber^7,8^, within the extracellular matrix (ECM), as well as changes in alveolar structure and reduction of inflatable lung units^9,10^. Such factors elevate lung stiffness, creating difficulties in physiological breathing and artificial ventilation^11–13^. The induced alterations increase the likelihood of further damage from overcompensation of non-fibrotic regions, causing progressive and irreversible lung injury^14,15^.

Mechanical strain informs our understanding of pulmonary behavior and function^7,16,17^ since lung injury is linked to elevated and heterogeneous dispersions of tissue strains^18,19^. Fibrotic lungs, specifically, are marked by spatially altered fiber deposition, resulting in abnormal lung deformations in comparison to healthy counterparts^20–22^. Studies have historically relied on bulk (global pressure–volume) lung mechanics, microanatomy, and computed tomography^5,11,23^ to estimate local behaviors^7^. Such investigations cannot characterize the real-time evolution of local tissue deformations over the breathing cycle. Additionally, these analyses are insufficient in characterizing nuanced and region-specific manifestations of disease, motivating the consideration of fibrosis attributes via local-level, time-continuous, and strain evolutionary analyses^24,25^.

Here we explore murine lung mechanics in fibrotic versus healthy specimens by assessing their local strain behaviors as influenced by continuous global pressure–volume measures for the first time. Our established custom-designed electromechanical ventilation system imposes global loads and collects bulk pressure–volume measurements^26,27^; the system is coupled with digital image correlation (DIC) technology to measure local tissue strains in response to various applied ventilation volumes and rates^28,29^. Use of DIC allows the novel connection of global to local behaviors, facilitates continuous measurement of temporally changing (evolutionary) tissue strain values, and measures full-field deformations to accurately assess tissue strain heterogeneity^30,31^. While local characteristics of fibrosis are often quantified via conventional biochemical and histological assays which demonstrated the altered matrix and fiber spatial arrangements^20,22^, here we consider the possibility of characterizing disease manifestation through local mechanics with sectioned regions of interest (ROI) on the lung surface. For both fibrotic and healthy pulmonary specimens, we characterize strain dispersions, lung distensibility, local and global compliance, rate dependencies and direction-dependent (anisotropic) behaviors and discuss possible disease-dependent changes in underlying mechanisms, such as alveolar recruitment. Additionally, this novel study evaluates strain evolution in diseased compared to healthy murine lungs by considering both whole-lung and region-specific trends. Thus, the novelty of this manuscript is to assess if the effects of these known tissue-level changes are detectable via local mechanics by use of strain measures. We provide a new perspective for understanding and evaluating the mechanics of fibrosis and enable a framework for extending this analysis to other pulmonary conditions. Additionally, given the relevance of murine lungs to human studies^32,33^, our examination of a clinically relevant disease model, wherein chronic inflammation arises from environmental factors and leads to untreatable fibrosis, may be helpful to inform human disease progression and clinical ventilation.

## Materials and methods

### Specimen procurement and preparation

**Figure 1:**
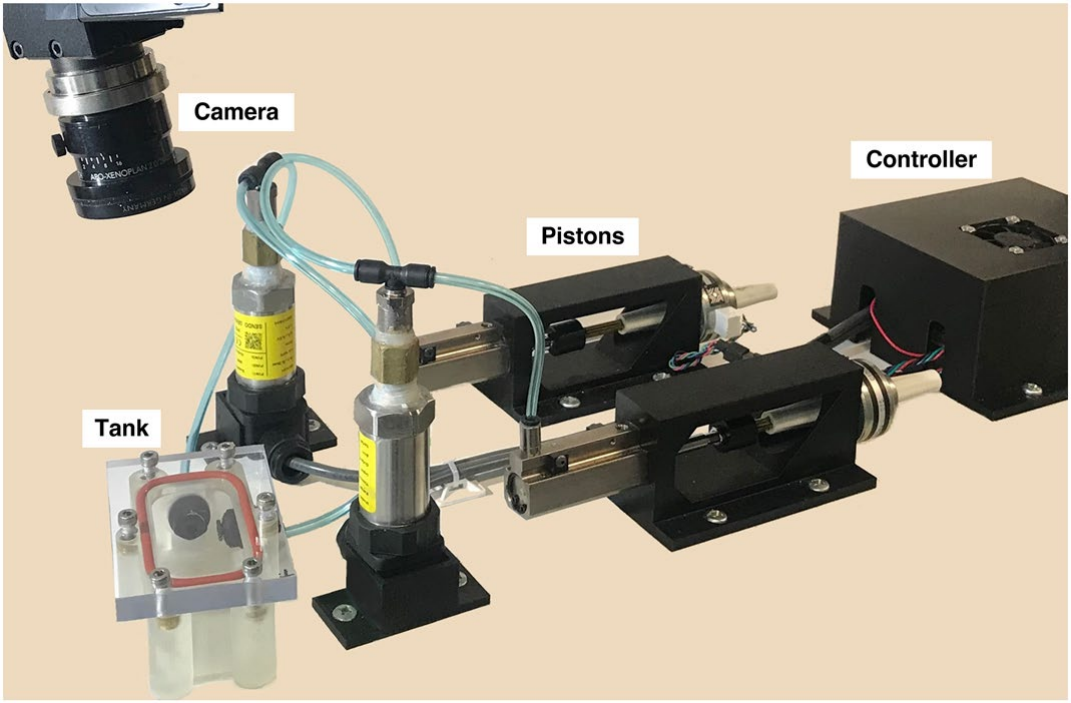
The test configuration is shown. Pistons provided continuous volume-controlled loads to inflate the lung test specimen, while accounting for tank air volume displacement to measure lung pressure and actual lung volume. Two overhead cameras recorded the lung specimen’s surface deformations in three dimensions to obtain DIC strain measurements.

Under approval of the University of California at Riverside (UCR) Institutional Animal Care and Use Committee (AUP#20200014), and following institutional guidelines and regulations, ten male C57BL/6 J mice (31.4 ± 3.5 g) were acquired from Jackson Laboratory (Bar Harbor, ME, USA) at 8–12 weeks old^34^. Mice were housed for 21 weeks in micro-isolator cages in UCR’s vivarium with unlimited food and water access. Physiological and behavioral patterns were monitored, and body weights were measured weekly. Over the 21 week period, mice were intranasally administered either 1X phosphate-buffered saline (PBS) or agricultural dust three-times weekly to serve as healthy controls or to model chronic obstructive pulmonary disease (COPD) manifested as peribronchiolar fibrosis leading to tissue fibrosis, respectively^6^. Post-treatment, mice were anesthetized and sacrificed by intranasal exposure to a cotton ball doused in 5 ml of isoflurane, and death was further verified via cervical dislocation and bilateral thoracotomy. Then, tracheal cannulation and lung extraction was performed. All procedures are reported in accordance with ARRIVE guidelines. Immediately following dissection, lungs were inflated via syringe to 0.5 ml to avoid airway collapse. Lungs were then prepared for ventilation and digital image correlation (DIC) data collection as detailed extensively previously^28,29^. Briefly, lungs were lightly airbrushed with white paint for contrast to the black randomized speckle pattern, which was applied via a fine-mist spray bottle, enabling continuous point displacement tracking for DIC^35^. Specimens were positioned beneath DIC cameras inside the tank of our custom-designed, dual-piston ventilation system, which accounts for air compressibility in real-time and reports actual lung change in volume  (Fig. 1)^26^. 1X PBS was used to maintain tissue moisture during testing. Specimens were ventilated and DIC data was collected concurrently, facilitating simultaneous measures of global pressures and volumes and local deformations and strains caused by lung expansion^28,29,36^.

### Test procedure and data analysis

The ventilation testing sequence began with a preload of 5 cmH_2_O to establish a common datum state and ensure comparable analyses^26,29^. The testing protocol included three inflation-deflation preconditioning cycles with a subsequent fourth cycle for analysis^37^. Volumes of 0.3, 0.5, and 0.7 ml were applied, each at rates of 5 and 20 breaths per minute (BPM). Lung tissue leaks are notoriously unavoidable as testing progresses, resulting in four viable control and three fibrotic specimens^16^. In agreement with former quasi-static studies^11,38^, frequencies were chosen to eliminate complex flow effects, while still maintaining physiological relevance^39,40^, as established previously^26,29^. DIC data was collected and analyzed as detailed in previous works^28,29^, briefly summarized below.

**Figure 2:**
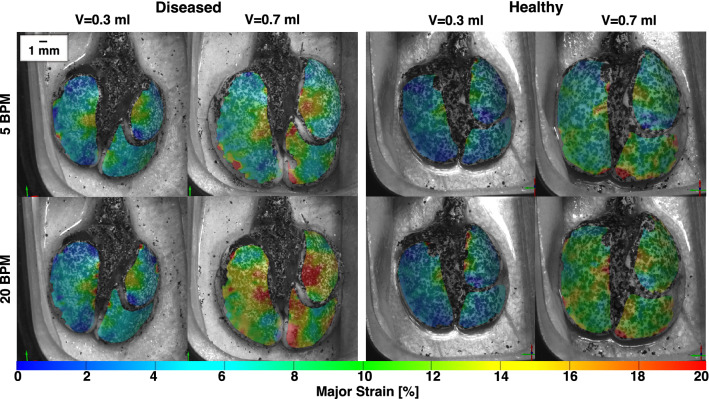
Representative murine diseased and healthy lungs topological major strain contours, shown at peak inflation for the smallest and largest applied volumes (0.3 and 0.7 ml) and the two inflation frequencies (5 and 20 BPM), illustrated qualitative strain variations between volumes, inflation rates, and health state.

**Figure 3:**
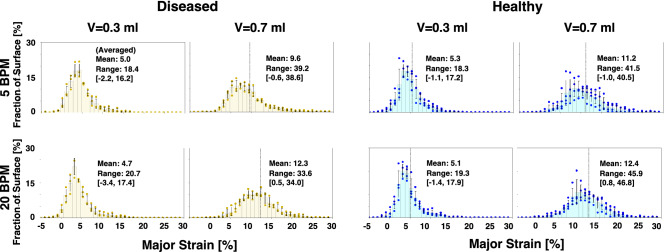
Diseased (gold) and healthy (blue) mouse whole lung strains obtained via DIC across specimens (average ± standard deviation of the individual mice values shown). The histograms depict the fraction of the surface of which strain values occurred, for applied volumes of 0.3 and 0.7 ml, and at the two inflation frequencies of 5 and 20 BPM. In addition, the whole lung surface’s mean and range strain values are included.

DIC deformation data was used to compare the diseased versus healthy lung surface tissue response by assessing local strains. Whole lung topological contour maps of principal major technical strain^41^ at end-inspiration (i.e., peak inflation) were used to qualitatively evaluate strains (Fig. 2). Strain distributions were quantitatively assessed using histograms (Fig. 3). Strain range was used to analyze strain dispersions in diseased and healthy tissue^29^.

**Figure 4:**
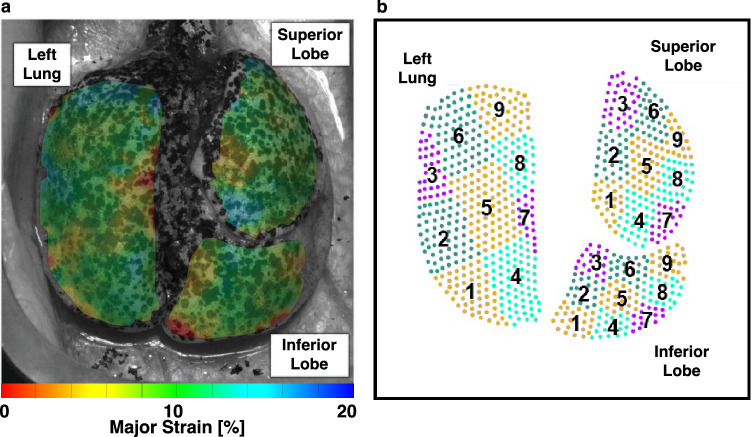
(**a**) Representative murine lung’s major strain contour map. Clockwise from top left, the left lung (LL), superior lobe (SL), and inferior lobe (IL) are labeled. (**b**) Each lobe’s corresponding regions of interest was illustrated and used to compare diseased and healthy specimens’ regional strains.

**Figure 5:**
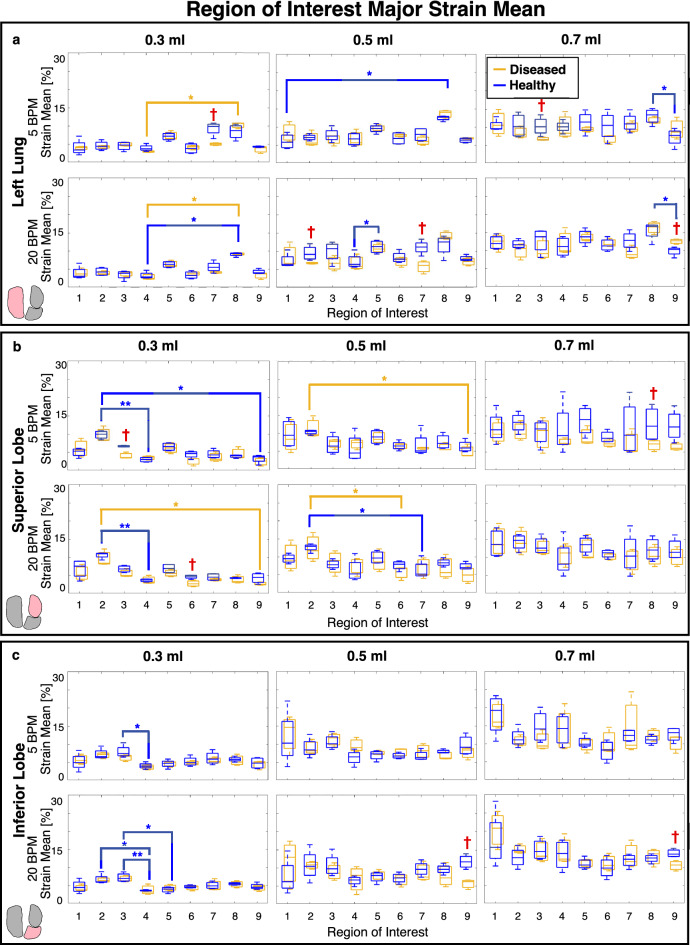
Diseased (gold) and healthy (blue) specimens’ peak inflation major strain mean values within each ROI for the (**a**) left lung, (**b**) superior lobe and (**c**) inferior lung surfaces, depicted via box and whisker plots. Applied volumes of 0.3, 0.5, and 0.7 ml at rates of 5 and 20 BPM are shown. Significant differences between diseased versus healthy ROI’s strain means are indicated with red daggers. Additionally, significant differences between each of the regions within diseased and healthy lungs’ groups are denoted by gold and blue asterisks, respectively.

**Figure 6:**
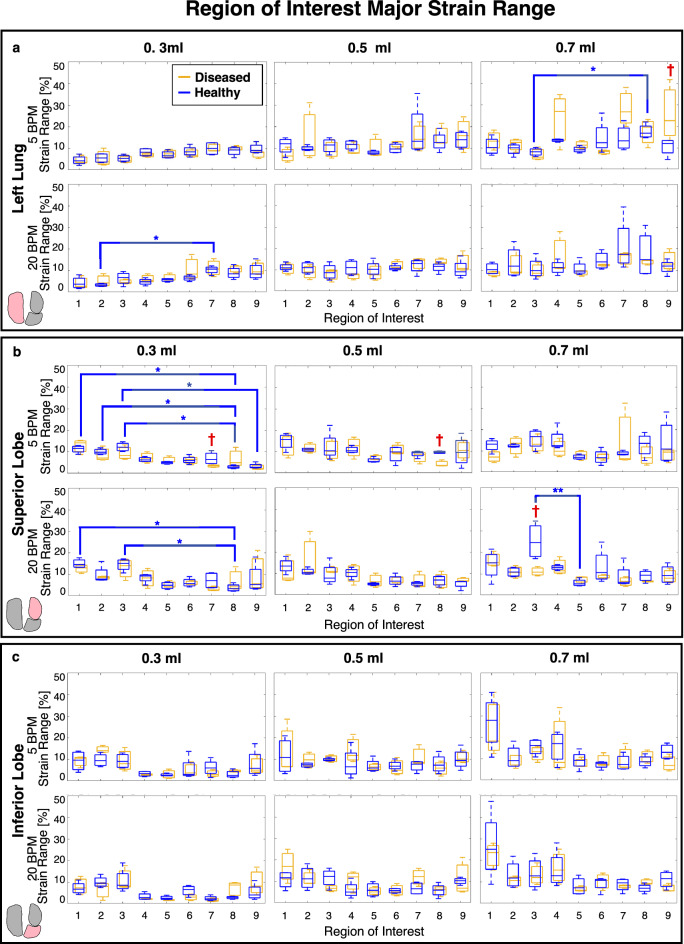
Box and whisker plot representation of diseased (gold) and healthy (blue) strain range within each ROI for the (**a**) left lung, (**b**) superior lobe and (**c**) inferior lobe, at each applied volume and breathing rate. Significant diseased versus healthy differences are indicated with red daggers, and significant differences between each of the regions within healthy lungs’ groups are denoted by blue asterisks. Intercomparisons of the diseased ROIs yielded no significant differences.

**Figure 7:**
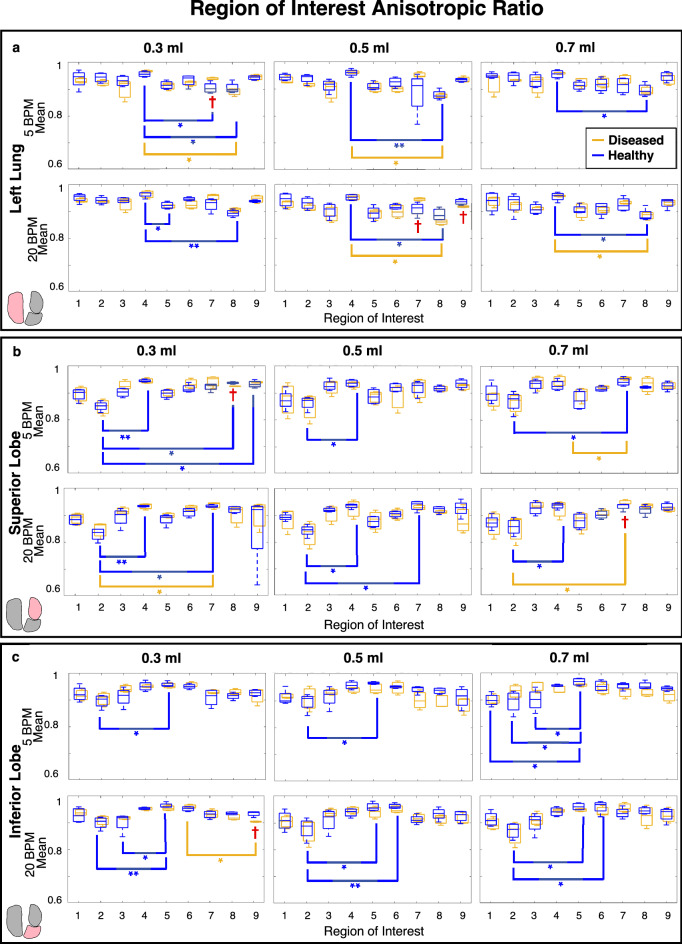
Box and whisker plot representation of diseased (gold) and healthy (blue) strain average anisotropic ratio for each ROI for the (**a**) left lung, (**b**) superior lobe and (**c**) inferior lobe, at each applied volume and breathing rate. Significant diseased versus healthy differences are indicated with red daggers, and significant differences between each of the regions within diseased and healthy lungs’ groups are denoted by gold and blue asterisks, respectively.

Spatially heterogeneous strain patterns motivated utilizing region of interest (ROI) analysis^42^ to delineate local manifestations and degree of strain in diseased compared to healthy lungs. An overlaid coordinate system was used to define the lungs’ ROI divisions and was associated with DIC data. Individual left lungs (LL), superior lobes (SL), and inferior lobes (IL) shown in Fig. 4a were partitioned into nine regions with three vertical divisions along the defined longest coordinate axis (cranial-caudal length) as the primary orienting axis, and three horizontal divisions of the shortest axis (medial–lateral length) as the secondary orienting axis (demonstrated by Fig. 4b). This segmentation method ensured position independence and equidistant ROIs regardless of specimens’ geometrical differences. Mean and range values of major strain and strain anisotropic ratio (defined as the ratio of minor to major strain) were evaluated within each of the nine regions at peak inflation (Figs. 5, 6, 7)^29,36^, and were compared between diseased and healthy specimens. Additionally, strain measures of each ROI were individually compared to other ROIs across the surface within the diseased and healthy groups for intercomparisons of potential heterogeneous lung expansion.

**Figure 8:**
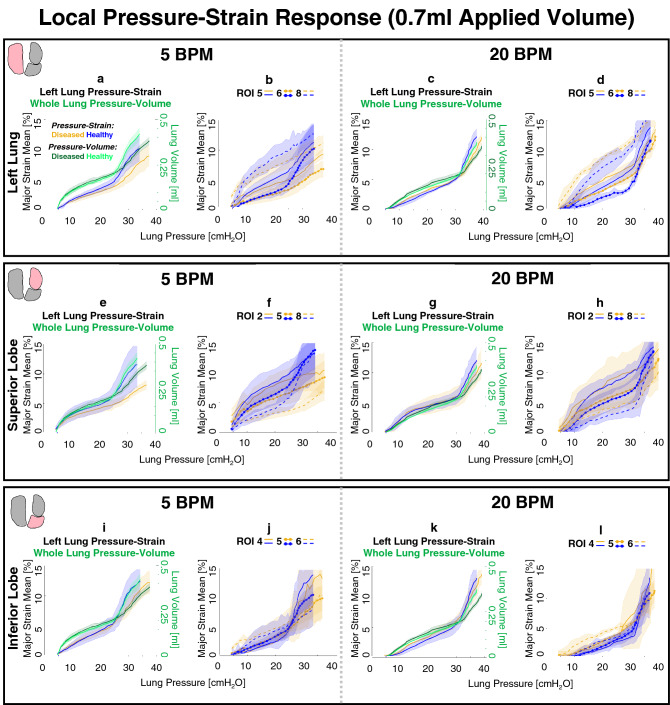
Local compliance pressure-strain curves for diseased and healthy murine lung specimens (gold and blue, respectively) compared to global lung pressure–volume compliance curves (green) and select ROIs of each lobe at both breathing rates (5 and 20 BPM), for an applied inflation volume (0.7 ml), for the left lung (**a**–**d**), superior lobe (**e**–**h**), and inferior lobe (**i**–**l**). Curve shadows represent the average ± standard deviation across specimens in the diseased or healthy groups.

Global compliance was calculated as the slope of whole lung pressure–volume (PV) curves (shown at 0.7 ml applied volume in Fig. 8), found by fitting curves to a linear regression of R^2^ > 0.9 (MATLAB, MathWorks Inc., Natick, MA, USA) as demonstrated previously^27,29,43,44^. Curve bilinearity was not observed at the two lower applied volumes and thus only the initial slopes across volumes were compared between diseased and healthy lungs for 0.3 and 0.5 ml^27,29^. Initial and final global compliance slopes were calculated for 0.7 ml, wherein bilinearity was consistent^45^; the pressure associated with the curve’s transition between initial and final slopes were computed^2,43^, and used to consider the recruitment of lung units^45–47^. To assess expansion capabilities, global peak inflation pressure and volume were compared between diseased and healthy specimens. To illustrate the distinctions between global and local behaviors, the individual lobe’s tissue strain evolution was also evaluated by determining the local initial and final compliance slopes of pressure-strain curves for 0.7 ml. These local compliance curves were compared between diseased and healthy lung tissue. Additionally, select ROI’s pressure-strain slopes were evaluated to further illustrate qualitative differences of region-specific tissue-level compliance.

### Statistical evaluations

In congruence with the sample size, non-parametric statistical analyses were appropriately chosen^48^. For each rate and volume, the two-tailed Mann–Whitney test was used to compare diseased versus healthy specimens’ peak inflation tissue strains for the whole lung (Fig. 3), and within each ROI (Figs. 5, 6, 7). Mann–Whitney was also used to compare whole lung pressure–volume curves’ slopes, transition pressures, maximal pressures and volumes, as well as local lobe pressure-strain curves’ slopes and transition pressures (Fig. 8). The two-tailed Wilcoxon test quantified effects of rate on whole lung averaged tissue strains (Fig. 3), local pressure-strain slopes, and transition pressures (Fig. 8). For both Mann–Whitney and Wilcoxon tests, significance was determined at an adjusted p-value of ^†^p < 0.06 (as required by the two study groups^48,49^). Intercomparisons of ROIs’ peak inflation strains was conducted via the Friedman test with Dunn’s post-hoc analysis for multiple comparisons (Figs. 5, 6, 7). Friedman’s test followed by Dunn’s was also used to assess volume comparisons of peak inflation whole lung strains (Fig. 3). Significance levels for Friedman’s and Dunn’s test were denoted by *, **, and ***, for p < 0.05, p < 0.01, and p < 0.001, respectively.

## Results

Heterogenous surface strain dispersions were qualitatively observed for both diseased and healthy specimens (Fig. 2). Spatially non-uniform strains were exacerbated with increasing volume and rate. Heightened strain often appeared in the medial region of the LL and SL and the distal region of the IL for both treatment groups, and at greater magnitudes in diseased states.

Lung strains were quantified and average strains of the whole murine lung surfaces trended greater in healthy compared to diseased specimens (Fig. 3). Mean strain unidirectionally increased with increasing applied volume for both diseased and healthy specimens at both rates. Specifically, significant increases within both treatment groups occurred when comparing the lowest (0.3 ml) to the highest (0.7 ml) applied volume (p < 0.05). The 5 BPM frequency for the diseased group, however, showed the smallest gain in strain magnitude when comparing the smallest and largest applied volumes. Comparisons of strain means between the two frequencies yielded the following trends: in both diseased and healthy specimens, the slower rate (5 BPM) revealed higher strain mean at the lowest volume (0.3 ml) compared to 20 BPM, while the faster rate (20 BPM) yielded greater mean strain at the two greater volumes (0.5 and 0.7 ml) compared to 5 BPM.

Generally, strain range was slightly greater in diseased compared to healthy specimens for applied volumes of 0.3 and 0.5 ml, at both frequencies. However, for the largest volume of 0.7 ml, healthy specimens yielded greater strain range values compared to diseased, significant at 5 BPM (p < 0.06). Similar to strain mean, increasing volume yielded a unidirectional increase of the strain range, with significant differences observed in healthy specimens (but not diseased state) between 0.3 and 0.7 ml for both frequencies (p < 0.05). Neither treatment group showed significant differences in the strain range between rates.

Figure 4a shows a representative strain contour of mice lungs with a non-uniform deformation profile quantified via nine ROIs segments (Fig. 4b) for localized analyses of strain data and comparisons between diseased and healthy states.

Comparisons between the major strain means within corresponding ROIs of diseased and healthy lungs yielded significant differences within the various applied volumes and frequencies (Fig. 5). Strain means within the ROIs of diseased specimens were generally lower than those of healthy counterparts for each lobe. For both rates, this trend was rather consistent across the various volumes and was particularly pronounced for the greatest inflation volume of 0.7 ml at 5 BPM. Additionally, statistical intercomparisons of lung surface ROIs showed a tendency for greater regional variability in healthy lungs, particularly at the lowest volume of 0.3 ml, compared to diseased specimens. Healthy specimens showed more interspecimen spread compared to diseased subjects.

Comparisons of strain range between diseased and healthy lungs’ corresponding ROIs yielded significant differences (Fig. 6). Diseased specimens tended to yield lower strain range amongst regions than healthy counterparts for 5 BPM. Conversely, at 20 BPM, the healthy group typically exhibited greater range of strain values compared to diseased specimens, primarily for 0.7 ml. Additionally, direct ROI to ROI comparisons found that several healthy specimens yield statistically significant different range of strain between regions, while diseased specimens yielded none. The SL showed the most regional variability.

Diseased versus healthy ROI comparisons for the strain anisotropic ratio resulted in limited significant differences for any applied volume and rate combination (Fig. 7). At both rates, diseased specimens tended to yield lower anisotropic ratio values within each lobes’ ROIs compared to healthy counterparts, particularly at 0.5 and 0.7 ml for the faster breathing frequency (20 BPM). Additionally, all anisotropic ratio values were above 0.8 with a majority closer to 0.9 – indicating near isotropic tissue stretch. Lastly, intercomparisons of regions’ anisotropic ratios revealed that healthy specimens showed many instances of significant variability between ROIs (particularly for IL) compared to fewer instances in diseased lungs.

Diseased and healthy lung specimens’ averaged whole lung-level, lobe-level, and select ROI-level pressure-strain evolutionary responses are depicted in Fig. 8 (for an applied volume of 0.7 ml and both rates) to investigate contrasts between local and global compliance and recruitment behaviors. Pressure-strain curves’ local initial and final compliance slopes, as well as transition pressures, were compared between diseased and healthy specimens at the lobe-level (Fig. 8a,c,e,g,i,k) and notable trends were observed: initial lobes’ local compliance slopes were consistently smaller for diseased compared to healthy lungs (not significant). While comparisons of local initial compliance between the rates yielded no significant differences for either treatment group, local initial compliance slopes were always lower for 5 compared to 20 BPM for each lobe of the diseased group. Conversely, healthy lungs (i.e., SL and IL) tended to show greater local initial compliance slopes for 5 in comparison to 20 BPM.

Local final compliances were also reduced for diseased lobes compared to those of healthy (not significant). Rate comparisons revealed lower local final compliance slopes trends for 5 compared to 20 BPM for each lobe of diseased and healthy lungs. In comparison to healthy lungs, diseased specimens’ lobes showed relatively large increases in the local final compliance slope value when the rate was increased from 5 to 20 BPM.

While not statistically significant, the transition between the initial and final portion of local compliance curves uniformly occurred at higher measured pressures for diseased lungs. While rate comparisons of slope transitions also yielded no significant differences for neither diseased nor healthy lobes, the transition tended to occur at higher measured pressures for 20 compared to 5 BPM.

The pressure-strain curves of diseased and healthy specimens at the ROI-level (Fig. 8b,d,f,h,j,l) qualitatively followed unique trajectories, indicative of region dependency.

Comparisons of diseased versus healthy lungs’ global compliance curves revealed that initial compliance slopes trended lower for diseased states at the lowest applied volume of 0.3 ml. As volume was increased to 0.5 and 0.7 ml, the initial compliance slopes tended to become more similar between diseased and healthy specimens. At 0.7 ml, however, the global final compliance slopes were typically greater for healthy lungs, with significance at 5 BPM (p < 0.06). Curve transition pressures tended to occur at higher measured lung pressures for diseased lungs.

For all volumes and frequencies, global lung response pressures and volumes were determined at peak inflation. While differences were non-significant between diseased and healthy lungs, diseased specimens tended to expand less and reach lower volumes in comparison to healthy specimens, particularly for 0.7 ml.

## Discussion

In this comprehensive study, we analyze the behavior of diseased compared to healthy murine lungs’ real-time ventilation evolution of local deformations for the first time via measures of deformation heterogeneity, anisotropy, and local and global compliance. A segmented (ROI) analysis of local tissue deformations is linked to global pressure–volume bulk behaviors, allowing region-specific, improved resolution, and more nuanced insights between diseased and healthy specimens. The main findings of this study are that the local and global trends of fibrotic lungs reflect their decreased distensibility, suggest altered recruitment behaviors, and show lessened adaptability. Relating the real-time evolution of local to global measures enables the identification of regions in the fibrotic lung that may be subject to overdistension, and how the deformation of these regions progress throughout inflation. Such findings may provide valuable characterizations of the diseased lung’s response to mechanical ventilation, which may be beneficial to understanding the development of VILI in fibrotic lungs undergoing clinical ventilation^15,50^. Furthermore, this study’s characterization of the continuous local, tissue-level, response to loading for the fibrotic lung in comparison to the healthy state provides a foundation to expand to this analysis to other diseases, such as asthma, emphysema, or COVID-19.

### Distensibility of diseased versus healthy lungs

The diseased lung’s mean surface strains are slightly less in magnitude than those of healthy (Fig. 3)^15^; this trend, while statistically insignificant, is in agreement with past works which find that fibrotic tissue is characterized by greater stiffness than healthy tissue (i.e., lower compliance)^9,10,13,20,50^, and exhibits areas of poor perfusion^51^, thus limiting expansion capabilities. Previous studies find reduced volume and increased stiffness for diseased lungs by use of global pressure–volume loops and histological data^11,22^. Congruently, we find global final compliance slopes of diseased lungs are often diminished (0.7 ml applied volume is shown in Fig. 8), in conjunction with trends (not significant) of decreased surface strains at peak inflation (Fig. 3). Additionally, our measurements of resultant global lung volumes show a tendency for lungs with fibrosis to reach smaller volumes than that of healthy lungs when delivered an equivalent amount of air, as seen in previous studies^9,12^. These findings, taken together, show how the well-regarded global evidence for decreased distensibility in diseased compared to healthy lungs may be similarly evident at the local level.

### Heterogeneity

Strain heterogeneity (Fig. 2) is delineated by ROI intercomparisons, and for healthy specimens we find many significant ROI versus ROI differences, versus fewer in disease (Figs. 5, 6). Interestingly, significant differences between regions of healthy lungs are more prominent at the 0.3 ml applied volume (Figs. 5, 6)—near the mouse’s physiological regime of tidal breathing^52^. Our findings may reflect the natural proclivity for heterogenous lung expansion (Fig. 2)^28^, which is altered (but not necessarily increased) in disease^53^. On the other hand, diseased lungs show lessened heterogeneity such that fewer regions are significantly disparate from one another (i.e., less ROI versus ROI differences) in comparison to healthy specimens (Fig. 5). Instead, for fibrotic lungs, distinct ROIs (i.e., the LL’s eighth and SL’s second ROI) show significantly elevated strains (Figs. 2, 5), clustering primarily in caudal regions—perhaps reflecting known fibrosis manifestations, which begin peripherally and progress inward^4^.

Fibrosis is marked by ECM abnormalities, with regions of exceptionally high collagen content that contribute to lung stiffening^7,9,13,20,22^. The spatial arrangement of scarred tissue adjacent to normal tissue, each with disparate mechanical stiffnesses, results in imbalanced expansion during inflation, with under-aerated regions adjacent to over-aerated areas^20,21,51^. Congruently, we see that fibrotic lungs show many regions with slightly less strain than healthy counterparts (Fig. 5), but exhibit particular areas where strain is elevated, likely where overdistention occurs (i.e., LL’s eighth, SL’s second, and IL’s first ROI; Fig. 2). This behavior may be a result of overcompensated expansion for underinflated regions, and such overstretched parenchymal strains are a main indicator of ventilator induced lung injury (VILI)^18^. The region-specific strain trends observed in this study can help to reveal areas susceptible to injury and further damage propagation^15,50^.

### Tissue compression

We observe that strains are not strictly in tension (positive strain), rather, compressed regions are seen at reduced ventilation volumes (negative strain, Fig. 3). Previous studies note restricted lung expansion and compressive deformations, and attributed this to positioning within the chest cavity^42,54,55^. Instead, we find negative strains even in the absence of the ribcage, in agreement with previous studies, which note that lung elastance is almost entirely attributed to parenchyma in mice^33,56^. We observe this compression for smaller ventilation volumes only, where airway recruitment is incomplete and expansion is limited, likely rendering inflated regions adjacent to uninflated regions, which undergo slight compression as a result (Fig. 2). These squished regions may indicate uneven alveolar ventilation, which is thought to be more present in abnormal lungs^57^; as such, we observe that at small inflation volumes, the strain ranges tend to be greater for diseased lungs at lower volumes, mainly due to the presence of more negative strain values (Fig. 3).

### Anisotropy

We quantify strain anisotropy and note that there is limited treatment group dependency. We assess average strain anisotropic ratios within each ROI of diseased and healthy murine lung parenchymal surfaces, and observe that diseased specimens’ ROIs exhibit a slight tendency (not significant) toward lower strain anisotropic ratio values than healthy lungs (Fig. 7), indicating greater distortion in fibrosis^58^.

Assessment of anisotropic, or direction dependent, characteristics may reveal abnormalities in lung function^59^. In human subjects, the degree of anisotropy is considered a potential biomarker for lung illnesses, with decreased anisotropy seen in advancing COPD^58,60^. Moreover, it is speculated that decreased anisotropic expansion is likely attributable to breakdown in lung structure and increased trapped air^58^. Air trapping is advanced by increasing inflation volumes and is predominantly increased with faster inflation rates^61^. Accordingly, we find the trend of decreased anisotropic ratios for diseased specimens and further note this occurs primarily for the faster rate and greater applied volumes of 0.5 and 0.7 ml (Fig. 7).

### Local and global evolutionary behaviors

Local pressure-strain curves (Fig. 8) are analyzed analogous to the conventional global pressure–volume curves to describe local level lung compliance^29,36^ and we calculate the slopes that distinguish the two inflation phases: local shallow initial and final steep compliance slopes. As in the case of global pressure–volume curves, these slopes are associated with the beginning of alveolar recruitment, followed by more substantial recruitment^46,47^. We find the slope transition occurs at higher measured pressures in diseased compared to healthy specimens (Fig. 8a,c,e,g,i,k), which may be attributable to changes undergone in alveolar structure in fibrosis^10,51,62^, such as inflammation, thickening, and decreased diameter^6,63^, in addition to increased parenchymal stiffnesses from changes of the ECM^12,13^, and decrease in inflatable units^9^.

In murine lungs, collateral ventilation is a secondary recruitment scheme wherein an additional set of alveoli abruptly “pop” open to accommodate large inflation volumes^64^. Previous studies show this is initiated at pressures of 20–25 cmH_2_O in murine lungs^27,45^. Similarly, we see an abrupt increase in tissue strain due to distension of the lower (caudal direction) periphery in diseased and healthy lobes, particularly for the IL, which generally occurs near the slope-transition pressure of 24.6–33.0 cmH_2_O (variable based on inflation rate). Expected differences are likely attributable to the disparate mice strain types (8 week old female BALB/c versus 8–12 week old male C57BL/6J mice used in this study) as well as changes resulting from lung extraction. The heightened strain is sustained throughout the remainder of inflation^29^, as seen in Fig. 2’s illustration of the lower IL at both frequencies and at 0.7 ml. Such trends are most likely ascribed to collateral ventilation, which we observe in healthy and diseased states alike. However, we also find this recruitment mechanism could be inhibited by changes undergone in fibrosis. Specifically, we observe the initial local compliance slopes are rather parallel between diseased and healthy lungs but diverge for the final local compliance (Fig. 8a,c,e,g,i,k), where the diseased state tends to have decreased slope values—a stiffer response—compared to healthy counterparts. The diseased lungs’ final local compliance slopes may illustrate specific alteration of the collateral recruitment phase, indicative of the fibrosis-induced alveolar-level changes (i.e., area reduction^6^), which may have negative implications for gas distribution during ventilation^51^.

ROIs facilitate the inspection of region-specific evolutionary deformation states between diseased and healthy lungs as it relates to the global bulk volume change level. The ROIs’ compliance curves demonstrate differing curve concavity trajectories from region to region (Fig. 8). The local ROI compliance curves are noted to exhibit disparities between the diseased and healthy specimens, often more pronounced than insights offered by the bulk behavior seen in the global compliance curve or lobular curves, where the strains are averaged across the entire lobe surface. Given the observed regional variability of the tissue, we underscore the potential value of inspecting the lung with more granularity for future studies.

### Rate considerations

Analyses of temporal dependencies stemming from the viscoelastic nature of lung tissue may offer crucial insights about varying loading conditions^13,39,65,66^, and in diseased and healthy lungs alike, the slower ventilation rate facilitates global and local compliance slope transitions at lower pressures compared to the faster rate (Fig. 8). This indicates how commencement of the secondary recruitment phase is enabled by slower breathing; as such, this may promote greater tissue relaxation, with more balanced gas distributions and alveolar opening^67^. Similarly, the slower rate appears to exhibit more homogeneous strain patterns, in agreement with previous studies ascribing maldistributions of gas to faster ventilation frequencies^19,68^. For diseased specimens in particular, the faster breathing rate tends to demonstrate elevated strain hotspots at the highest volume of 0.7 ml (Fig. 2). This has implications for clinical treatment of fibrotic lungs, where ventilation induces overwhelmingly negative outcomes^15,69,70^, but may improve with prudent use of protective strategies^71^.

## Limitations

Coupling DIC and ventilation yields valuable insights between local and global lung mechanics by directly measuring the region-dependent and time-continuous tissue response. However, this method is limited to directly linking the lung’s global pressure–volume measures to the lung’s topological (visible) surface, not its internal tissue deformations. While we are able to speculate associations between surface and internal mechanics levels—particularly for murine lungs, which exhibit a greater fraction of alveoli near the pleural surface in comparison to other species^59^—seeking real-time quantification of tissue deformations as yielded by DIC necessitates this tradeoff. Internal properties, such as gas distributions, can elucidate lung deficiencies^72^, and new techniques using dynamic imaging methods showcase the possibility of relating surface to internal deformations^63^. As such, future work will seek to directly associate surface to internal strains for our established ventilation protocols, which may be useful in fully understanding effects of various ventilation schemes, (i.e., correlations between slow rates and greater stress relaxation at the alveolar level^67^). This will help to better address clinically pertinent questions.

The well-established 21-week murine fibrosis model used in this current study was implemented in a previous study, with confirmed significant progression of fibrosis in this model using the Ashcroft’s score, as well as significant alveolar inflammation^6^. As such, although the heterogenous regional patterns we evaluated coincide with known manifestations of fibrosis disease, which begin in the alveoli (i.e., the periphery of the lung) and spreads inward^4^, we recognize these associations can only be speculated in the absence of explicit regional verification using techniques such as histology.

Presently, the findings yielded by these methods warrant removal of the lung from its physiological position in the chest cavity, potentially disregarding residual stresses and thus altering the tissue pressure-strain response^73^; however, it is worth noting that in mice the chest wall is shown to minimally contribute to respiratory mechanics in comparison to parenchyma^33,56^. To mitigate effects of ex vivo testing while maintaining physiological relevance, we test immediately upon removal, do not de-gas lungs, and aim to minimize disruption of surfactant^27^. We find that our three-dimensional surface strain values via DIC are decreased but on the same order of magnitude compared to those of in-situ methods using digital volume correlation (DVC) in rats’ lungs^16^; differences are expected due to the use of disparate species, preload, and methods of strain measurement. While use of DVC allows in-situ characterizations, such techniques require prolonged image acquisition time at discrete time stages making measures particularly susceptible to viscoelastic effects^63,74^; DIC methods do not share these constraints. It is important to note that the primary goal of this study is to establish baseline local mechanics of healthy versus diseased lungs and results should be cautiously extrapolated.

## Data Availability

The raw data supporting the conclusions of this article will be made available by the authors, without undue reservation.
